# Serum biomarkers, including nitric oxide metabolites (NOx), for prognosis of cardiovascular death and acute myocardial infarction in an ESSE-RF case–control cohort with 6.5-year follow up

**DOI:** 10.1038/s41598-022-22367-x

**Published:** 2022-10-28

**Authors:** Nadezhda G. Gumanova, Natalya L. Bogdanova, Victoria A. Metelskaya, Vladimir I. Tarasov, Alexander Ya. Kots, Vladimir A. Kutsenko, Anna V. Kontsevaya, Oksana M. Drapkina

**Affiliations:** Department of Biochemistry, National Research Center for Preventive Medicine (NRCPM), 10 Petroverigsky per., building 3, Moscow, 101990 Russian Federation

**Keywords:** Biochemistry, Biomarkers, Cardiology

## Abstract

The present case–control study aimed to assess associations of routine and experimental biomarkers with risk for cardiovascular death and acute myocardial infarction (AMI) in a cohort recruited from the multicenter study “Cardiovascular Epidemiology in Russian Federation” (ESSE-RF) to identify experimental biomarkers potentially suitable for expanded evaluation. A total of 222 subjects included cardiovascular death (N = 48) and AMI cases (N = 63) during 6.5-year follow up and matched healthy controls. Seven routine and eight experimental biomarkers were assayed to analyze associations with outcomes using logistic and Cox proportional hazard regressions. Elevated levels of cardiac troponin I (cTnI), C-reactive protein (CRP), and nitric oxide metabolites (NOx) were independently associated (P < 0.001) with higher risk of cardiovascular death (estimated hazard ratio (eHR) = 1.83–3.74). Elevated levels of NOx and cTnI were independently (P < 0.001) associated with higher risk of nonfatal AMI (eHRs = 1.78–2.67). Elevated levels of angiopoietin-like protein 3 (ANGPTL3) were independently associated (P < 0.001) with lower risk of cardiovascular death (eHRs 0.09–0.16) and higher risk of nonfatal AMI (eHR = 2.07; P = 0.01). These results indicated that subsequent expanded validation should focus on predictive impact of cTnI, NOx, CRP, and ANGPTL3 to develop nationwide recommendations for individual stratification of patients with cardiovascular risks.

## Introduction

Biomarkers are the indicators of the main pathological signaling pathways used to diagnose a disease and monitor the progression of the symptoms and efficacy of a therapy. Rational identification of biomarkers is based on pathology of specific diseases and associated factors^1^. Thus, pathology-related biomarkers are generally suitable for diagnostics and assessment of prognosis of various diseases^2^.

Biomarkers are characterized by specificity and selectivity. Diseases with simple pathological processes can be reliably assessed using a single biomarker. However, diseases with complex pathological processes may require multiple biomarkers, and risk of various acute events associated with these diseases or mortality usually requires assessment of a panel of factors. Diagnosis of cardiovascular diseases and prognosis or risk of acute events and mortality associated with these diseases typically involves assessment of multiple parameters. For example, evaluation of cardiovascular risk in Europe is based on SCORE (Systematic Coronary Risk Estimation) that includes evaluation of a combination of sex, age, systolic blood pressure, total serum cholesterol, and smoking status. SCORE or similar systems can be directly used for individual stratification of patients and are valuable tools for epidemiological assessment or general recommendations. However, SCORE is not suitable for stratifications of patients with previous acute cardiovascular events. Therefore, identification of a minimal panel of specific and selective biomarkers is required to enhance reliability of prognosis of acute cardiovascular events or cardiovascular mortality on individual basis.

Thus, we hypothesized that analysis of a panel of known experimental biomarkers in the serum may produce satisfactory predictions suitable for individual stratification of the patients based on the risk of acute coronary events or cardiovascular mortality. The panel included serum levels of 8 experimental markers: angiopoietin-like protein 3 (ANGPTL3), proprotein convertase subtilisin/kexin type (PCSK9), circulating total nitrate and nitrite ions (NOx), endothelin-1, leptin, adiponectin, galectin-3, and cardiac troponin I (cTnI). Most of these markers were not extensively validated but were previously demonstrated to be associated with cardiovascular risk in numerous studies and are directly or indirectly related to various aspects of cardiovascular pathology^3^. ANGPTL3 and PCSK9 participate in lipoprotein metabolism, which is associated with atherogenesis and development of cardiovascular lesions^4,5^. Leptin and adiponectin are the key regulators of energy metabolism. Serum level of NOx is a parameter used for indirect evaluation of homeostasis of nitric oxide (NO), a vasoactive mediator promoting relaxation of blood vessels that is also linked with inflammation, whereas endothelin-1 is one of the main vasoconstrictory mediators. Galectin-3, a β-galactoside-binding protein, and cTnI, a miofibrillar protein, are extensively used to characterize the presence and extent of cardiac injury in routine clinical practice and are officially approved by the US Food and Drug Administration^6,7^.

The aim of the present preliminary assessment was to determine suitable candidates for investigation of a panel of prospective biomarkers based on 6.5-year follow up of nonfatal acute myocardial infarction (AMI) and cardiovascular death in a relatively small case–control multicenter cohort in Russian Federation recruited from the large study “Cardiovascular Epidemiology in Russian Federation” (ESSE-RF). Health care and individual stratification in Russian Federation, similar to other large countries, faces considerable challenges due to insufficiently universal recommendations and specific guidelines for cardiovascular diseases. These problems are mainly caused by overwhelming variability introduced by multiple factors, including climate zones, lifestyle, nutritional habits, ethnicity, social status, and local economic conditions documented in multiple reports. Thus, pilot and proof-of-principle investigations for identification of universally suitable biomarkers should be based on a case–control design to limit at least some of this variability to enable acquisition of meaningful results using limited resources. This approach was previously used in case–control studies on troponin T to demonstrate associations with long-term mortality^8^. Specifically, we assessed predictive power for the outcomes of a small panel of biomarkers adjusted for sex and age and evaluated appropriate statistical models and required parameters for a planned subsequent large-scale prospective study.

## Methods

### Patients

The cohort used in the present study has been selected from the large cross-sectional multicenter population-based ESSE-RF study registered at ClinicalTrials.gov as NCT02449460 (2015–2016). The design of the whole ESSE-RF study and some preliminary results were published previously^9,10^. Briefly, the ESSE-RF study included 12 regions of Russian Federation and a 6.5-year follow up period. ESSE-RF used a multi-stage clustered sample design based on district outpatient departments (polyclinics) that were selected randomly as the primary sampling units. These primary sampling units covered local populations of approximately 30,000–80,000 adult residents. The following regions from 8 large Federal Districts of Russian Federation were involved in the ESSE-RF study: North Ossetia (Alania) Republic (North Caucasus Federal District), Volgograd (South Federal District), Vologda (North-West Federal District), Voronezh (Central Federal District), Ivanovo (Central Federal District), Kemerovo (West Siberia Federal District), Krasnoyarsk (East Siberia Federal District), Orenburg (Volga Region Federal District), Vladivostok (Far East Federal District), Saint Petersburg (North-West Federal District), Tomsk (West Siberia Federal District), and Tyumen (Ural Federal District). Participants were recruited by telephone calls and were asked to visit the clinic in the morning after overnight fasting. All participants signed a written informed consent before the enrollment. Trained staff at the participating cites conducted all interviews and collected the measurements. The interview questionnaire included the history of preexisting diseases: AMI, ischemic heart disease, arrhythmia, other cardiovascular diseases, diseases of the liver and kidney, cancer, and diabetes mellitus type 2. Questionnaire also included smoking status: never smoked (1), quitted (2), and actual smoker (3). The blood samples and data were collected from 2013 to 2015 according to the approval by the Independent Ethics Committee of the Federal State Budgetary Institution "NRCPM" of the Ministry of Health of Russia (approval no. 03-06/13 of 18.04.2013). The study was approved by three ethics committees, starting from 2011: National Research Center for Preventive Medicine (NRCPM; 8 participating centers), Russian Cardiology Research-and-Production Complex (2 participating centers), and Federal Almazov North-West Medical Research Centre (2 participating centers)^9–12^. After all approvals were obtained from all participating centers, the whole ESSE-RF study was registered at ClinicalTrials.gov in 2015. All procedures were compliant with the guidelines of the Helsinki Declaration and WHO for similar studies^9–12^.

**Figure 1:**
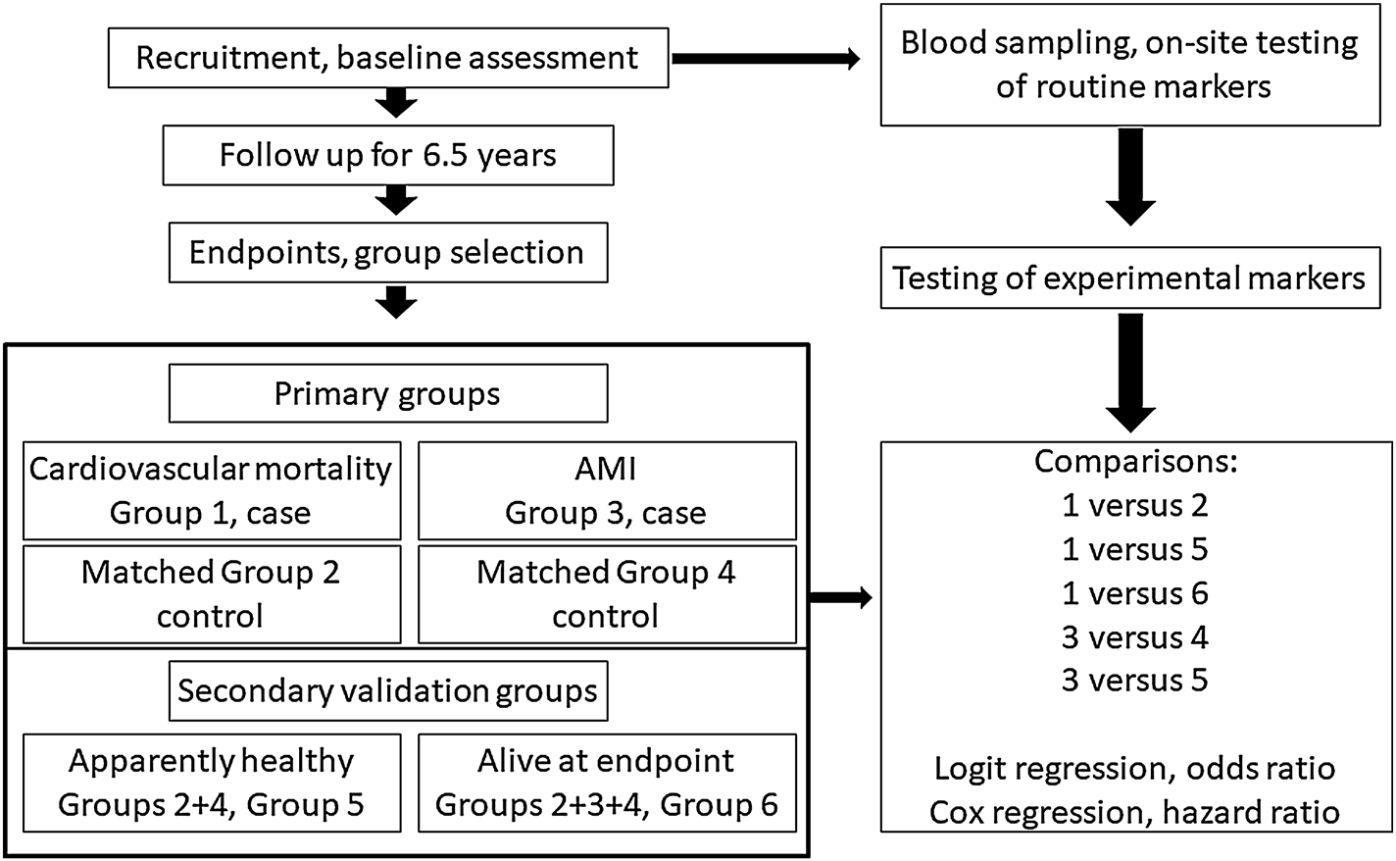
The workflow of analysis. Notable, group 2 was selected as individual matches to group 1, and group 4 was selected as individual matches to group 3 according to the criteria described in the text in detail.

The cohort used in the present case–control study for evaluation of predictive power of biomarkers included a total of 222 subjects of both sexes (111 cases and 111 controls; 134 male and 88 female subjects). The subjects were selected based on availability of the endpoint data and of the corresponding matched apparently healthy individuals (Fig. 1).

The number of selected subject was limited by available funding for reagents and supplies. The endpoints included cardiovascular mortality during 6.5-year follow up (N = 48; 77% fatal AMI and 23% other cardiovascular mortality; 30 male and 18 female patients) and nonfatal AMI (N = 63; 37 male and 26 female patients) in 7 regions included in ESSE-RF. Matching controls of the same sex were randomly selected from the corresponding regions to ensure mandatory similarity of biometric and demographic parameters as follows: (1) lack of an endpoint (cardiovascular mortality or nonfatal AMI); (2) survival to the end of follow up; (3) lack of diabetes mellitus, oncological diseases, arthritis, ischemic heart disease, arrhythmia, and other cardiovascular diseases; (4) age difference with the matching case no more than 2 years; (5) matching region, sex, and level of education. Additional non-mandatory matches were based on availability of the subjects and included smoking status (never smoked (1), quitted (2), actual smoker (3)), alcohol consumption, and body mass index. Specific inclusion criteria at enrollment included glomerular filtration rate of at least 60 mL/min evaluated according to the Cockroft-Gault equation based on the serum creatinine level to ensure compliance of all medical centers participating in the study. The serum and plasma samples collected during the enrollment period were stored in the Biobank of NRCPM, Moscow. Cause of death was confirmed based on official death certificates. Nonfatal AMI was confirmed by patient history files and the data of electrocardiography.

### Blood sampling

Blood was sampled from the cubital vein after approximately 12-h fasting. The serum and plasma were prepared on site, aliquoted, shipped, and stored in the Biobank of NRCPM at – 70 ºC until assay.

### Routine biochemical tests

Most of routine tests were performed locally in participating regional diagnostic centers according to the original design of ESSE-RF aiming to provide sufficient standardization of the tested parameters^9^ using specific guidelines approved by Center for External Quality Control of Clinical Laboratory Testing of Russian Federation (www.fsvok.ru). Some preliminary data describing routine test results have been published previously^9–12^. Total cholesterol (mM) and triglycerides (mM) were assayed on a Konelab 20i (Thermo Electron) automatic analyzer using kits from Human, Germany. High density lipoprotein (HDL) cholesterol (mM) was assayed after precipitation of low and very low density lipoproteins. The level of low density lipoprotein (LDL) cholesterol (mM) was calculated according to the Friedwald equation in the samples with serum triglycerides below 4.5 mM. Glucose (mM) in the plasma (hexokinase method, according to WHO recommendation, 2006) was assayed by kits from DiaSys (Germany) using a Sapphire-400 automatic analyzer (Japan). C-reactive protein (mg/L) was assayed by high sensitivity quantitative immunoturbidimetric method enhanced with latex particles (universal range 0.3–350 mg/L, highly sensitive 0.05–20 mg/L) at a wavelength of 800 nm (505 nm) using kits from DiaSys (Germany) by a Sapphire-400 automatic analyzer (Japan). Creatinine was assayed by the Jaffe method using DiaSys kits according to the recommendations of the Clinical Laboratory Standards Institute (CLSI) (document EP17-A) using a Konelab analyzer (Thermo Fisher Scientific, USA). Insulin (µIU/mL) was measured by an immunoassay kit (Abbott Diagnostics, USA).

### Experimental biomarker assays

All assays of biomarkers of this group were performed in NRCPM using local samples and samples shipped from the participating regions and stored in the Biobank. cTnI was measured in the serum by two-step high-sensitivity chemiluminescent microparticle immunoassay (detection limit 0.7–1.3 pg/mL; limit of quantification 10 pg/mL; CV% < 10%; Abbott Diagnostics, USA) using an Architect i2000SR analyzer (Abbott Laboratories, Ireland). Leptin was assayed using a sandwich ELISA kit from Diagnostics Biochem Canada, Inc., Canada (detection limit 0.5 ng/mL; CV% 5.8–6.8%); interleukin-6 was assayed using an ELISA kit from Affymetrix Bioscience, USA (detection limit 0.92 pg/mL; CV% 5.2); endothelin-1 (1–21) was assayed using a Quantikine ELISA kit from R&D Systems, USA (detection limit 0.04 pg/mL; CV% 5.2); adiponectin and PCSK-9 were assayed using ELISA kits from BioVendor, Czech Republic (detection limits of 26 and 9 ng/mL; CV% 6.3–7.0% and 7.5%, respectively); ANGPTL3 was assayed using an ELISA kit from RayBiotech, USA (detection limit 8.2 pg/mL, CV% 12%); galectin-3 was assayed using an ELISA kit from Invitrogen, Thermo Fisher, USA (detection limit 0.29 ng/mL; CV% 7.5%). Optical density was measured by a microplate reader (Infinite 200 PRO, Tecan, Switzerland). The concentrations of nitrate and nitrite (NOx) were measured in the serum deproteinized by ultrafiltration through Spin-X UF concentrators (Corning, England) using Griess reaction after reduction with vanadium (III) chloride^13^ as described previously^14,15^. Reagents for NOx assay were from Sigma-Aldrich (St Louis, MO, USA); the same lots of the reagents were used throughout all assays (detection limit 4 µmol/L, CV 10–15%)^15^.

### Statistical analysis

Statistical evaluation was performed using SAS software version 9.4 (SAS Institute, Inc., USA) and SPSS version 23 (IBM, USA). Normality of distribution was tested by Kolmogorov–Smirnov test. The data are shown as the median, minimal, and maximal values. Univariate logistic regression analysis used a binary logit model and Fisher scoring. Stepwise proportional Cox regression was evaluated based on the Akaike information criterion (AIC) and Schwartz criterion (SC) as estimators of prediction error and relative quality of statistical models. The assumption of proportionality of the Cox model was confirmed by independent Kaplan–Meier survival analysis. The models with the lowest AIC and SC values were selected for subsequent analysis. Multivariate proportional Cox regression was adjusted for covariates and evaluated by a chi-squared test to generate the Pr > ChiSq values for all models, which corresponded to the probability that at least one regression coefficient of a model is not equal to zero. Application of Cox regression to case–control studies is known to introduce certain bias in accurate assessment of the hazard ratios^16^. However, the main goal of the present study was to compare various markers. Thus, we generally assumed that the biases of various Cox models are similar. Moreover, the results of Cox models generally agreed with the results obtained by logistic regression and provided estimated dynamic assessment. Hence, the data of Cox regression analysis are presented as estimated hazard ratios (eHRs), which may be different from the true hazard ratios due to unknown bias. Moreover, we have performed internal confirmatory analysis, which could have reduced possible bias for the assessment. Considering overall simple design of the study, these assumptions are presumed to provide sufficient accuracy for the purposes of biomarker comparison^17^. Receiver operating characteristic (ROC) analysis was initially evaluated by the Hosmer-Lemeshov criteria to ensure an adequate fit. *P-*values < 0.05 were considered significant.

### Ethics declaration

The ESSE-RF study was approved by three ethics committees: National Research Center for Preventive Medicine (NRCPM; 8 centers), Moscow, Russia; Russian Cardiology Research-and-Production Complex (2 centers), Moscow, Russia; and Federal Almazov North-West Medical Research Centre (2 centers), St Petersburg, Russia. All procedures were compliant with the guidelines of the Helsinki Declaration and WHO for similar studies. All participants provided their written informed consent for participation in the ESSE-RF study.

## Results

### General characterization of the cohort and grouping

A total of 222 case–control matched participants (134 men and 88 women) were selected from the parental ESSE-RF cohort as described in the Materials and Methods section. The case subjects (N = 111) included 48 cardiovascular deaths (due to fatal AMI accounting for 77% of deaths and due to other cardiovascular diseases accounting for 23% of deaths; 62.5% men) and 63 cases of nonfatal AMI (58.7% men) detected during follow up of 6.5 years. Cardiovascular deaths and nonfatal AMI were significantly associated with pre-existing cardiovascular diseases diagnosed during the initial examination, including pre-existing AMI, ischemic heart disease, arrhythmia, and other cardiovascular diseases (data not shown). These associations may directly or indirectly account for mortality and nonfatal AMI. A total of 111 essentially healthy control subjects were manually pairwise selected from the parental ESSE-RF cohort to match the baseline demographics and other parameters of the case subjects (Fig. 1). The events detected in the control groups were not associated with baseline diseases indicated for the case subjects thus indirectly confirming apparently healthy baseline conditions of the control groups. Baseline clinical and demographic characteristics of the groups are listed in Supplemental Table S1. The age of the participants ranged from 36 to 65 years (a median of 57 years). Case–control matches were confirmed by identical values of demographic characteristics (Supplemental Table S1).

The cohort was used to evaluate the associations of 7 routine and 8 experimental biomarkers with risks for cardiovascular death and nonfatal AMI. Seven biomarkers are routinely used in clinical practice and have been validated in multiple studies, including CRP, creatinine, total cholesterol, LDL cholesterol, HDL cholesterol, glucose, and insulin. Additional 8 biomarkers were considered experimental for the purposes of the present study, even though cTnI has been previously shown to be associated with mortality in a number of studies^18^. These experimental biomarkers included leptin, adiponectin, endothelin-1, PCSK9, galectin-3, ANGPTL3, NOx, and cTnI^19^. Inclusion of cTnI enabled to confirm potentially useful experimental markers by direct comparison and provided overall quality control of the results of regression analysis.

The whole study cohort was divided into 4 main groups as shown in Table 1.

**Table 1:** Number of participants in the case and control groups used for model assessment.

Groups and events	Men, N	Women, N	Men and women, N
**Main groups**			
1, case, cardiovascular death	30	18	48
2, control, matched with group 1	30	18	48
3, case, nonfatal AMI	37	26	63
4, control, matched with group 3	37	26	63
**Additional groups**			
5, apparently healthy, groups 2 and 4 combined	67	44	111
6, alive at endpoint, groups 2, 3, and 4 combined	104	70	174
Total cohort	134	88	222

AMI, acute myocardial infarction.

The main groups included group 1 (cardiovascular deaths, case), group 2 (matched control to group 1), group 3 (nonfatal AMI, case), and group 4 (matched control to group 3). Two additional groups 5 and 6 were defined to facilitate the comparison and provide for internal confirmation of the results of regression analysis. These additional subgroups included group 5 (apparently healthy participants of group 2 and group 4 combined) and group 6 (all participants alive at the endpoint of the follow up period of groups 2, 3, and 4 combined). The main approach to the optimization involved initial comparison of the case groups with the corresponding control groups (group 1 versus group 2 and group 3 versus group 4) with subsequent internal confirmation of identified associations by comparing group 1 versus group 6 and group 3 versus group 5. The workflow of analysis is shown in Fig. 1.

### Univariate associations of biomarkers with cardiovascular deaths

All 15 biomarkers (routine and experimental) were assayed in the samples of all participants, and the results are presented in Supplemental Table S2, which also lists the corresponding reference values for routine biomarkers for moderate cardiovascular risks^19^. However, experimental biomarkers do not have certified and validated reference values.

All biochemical parameters were divided into terciles. Associations of the upper tercile (higher 33% values above the cutoff are shown in Supplemental Table S3) with cardiovascular death and nonfatal AMI endpoints were compared with those for the two lower terciles combined. Initially, comparison was performed using univariate logistic regression for group 1 versus group 2, group 1 versus group 5, group 1 versus group 6, group 3 versus group 4, and group 3 versus group 5 (Fig. 1, Supplemental Tables S4 and S5).

The results of this univariate logistic analysis indicated that six biomarkers were associated with cardiovascular deaths, including 5 biomarkers associated with an increase in mortality (cTnI, galectin-3, NOx, CRP, and creatinine) and HDL cholesterol associated with a decrease in mortality. The levels of cTnI selected as a reference were clearly associated with mortality regardless of sex, and the associations remained significant in all three tested comparisons (Supplemental Table S4). Galectin-3, CRP, and HDL cholesterol had significant associations with mortality in comparison of group 1 versus group 2 in men.

Internal confirmation by comparison of group 1 with group 5 indicated that these 3 biomarkers (galectin-3, CRP, and HDL cholesterol) preserved their associations. Moreover, two additional associations were detected, including ANGPTL3 и NOx, which were not sex-dependent similar to cTnI and unlike CRP, creatinine, and HDL cholesterol. Higher levels of ANGPTL3 were associated with a decrease in mortality similar to associations detected for HDL cholesterol.

Subsequent expansion of internal confirmation by comparison of group 1 versus group 6 indicated that NOx was no longer associated with mortality (Supplemental Table S4) apparently due to the impact of NOx associations with nonfatal AMI (Supplemental Table S5). However, ANGPTL3 and cTnI maintained the associations regardless of sex, and CRP maintained the association in men. Creatinine lost its association (Supplemental Table S4) apparently due to normal glomerular filtration rate, which was within the reference range for all subjects.

### Univariate associations of biomarkers with nonfatal AMI

Associations of biomarkers with nonfatal AMI were analyzed by comparisons of group 3 versus group 4 and group 3 versus group 5 (Fig. 1, Supplemental Table S5). Case–control comparison of group 3 versus group 4 indicated significant and sex-independent associations with NOx and associations with cTnI and insulin only in female subjects, although cTnI preserved the associations in the combined male and female subgroups.

Internal confirmation of these associations by expanded comparison of group 3 versus group 5 confirmed sex-independent associations of nonfatal AMI with NOx and association with cTnI in the female subgroup. This expanded comparison revealed sex-dependent associations of endothelin-1, galectin-3, and ANGPTL3 with nonfatal AMI.

Thus, the results of univariate analysis indicated that only elevated NOx was sex-independently associated with nonfatal AMI with an average odds ratio of approximately 3 (P < 0.03), and elevated ANGPTL3 was associated with nonfatal AMI only in men (Supplemental Table S5). Elevated levels of cTnI, galectin-3, and insulin were associated with a higher risk of nonfatal AMI predominantly in women. Moreover, cTnI, galectin-3, ANGPTL3, and NOx were also sex-independently associated with cardiovascular death.

### Stepwise Cox regression analysis

Cox regression was used to expand the description of the associations of biomarkers by inclusion of the time variable. Stepwise analysis was used to select optimal models (Table 2).

**Table 2:** Stepwise Cox regression analysis of associations of biomarkers with cardiovascular death and nonfatal AMI.

Comparison	Sex	Model	eHR	95% CI	Pr > ChiSq
**Cardiovascular death**					
Group 1 versus group 2	Men	cTnI	3.01	1.42–6.37	0.01
		CRP	3.19	1.53–6.64	0.01
	Women	cTnI	3.00	1.17–7.68	0.02
	Men and women	PCSK9	0.52	0.29–0.96	0.04
		Galectin-3	1.90	1.06–3.42	0.03
		cTnI	2.81	1.56–5.05	0.01
Group 1 versus group 5	Men	ANGPTL3	0.14	0.03–0.62	0.01
		cTnI	4.34	2.04–9.24	0.01
		CRP	4.13	1.94–8.81	0.01
	Women	cTnI	3.44	1.35–8.74	0.01
	Men and women	ANGPTL3	0.23	0.07–0.76	0.02
		cTnI	3.35	1.87–6.00	< 0.0001
		CRP	1.97	1.11–3.48	0.02
Group 1 versus group 6	Men	Endothelin	0.33	0.14–0.77	0.01
		Galectin-3	2.12	1.01–4.46	0.05
		ANGPTL3	0.10	0.02–0.42	0.01
		cTnI	3.58	1.65–7.76	0.01
		CRP	3.59	1.68–7.68	0.01
	Women	ANGPTL3	0.10	0.01–0.72	0.02
		cTnI	3.06	1.20–7.80	0.02
	Men and women	Endothelin	0.48	0.23–0.97	0.04
		ANGPTL3	0.13	0.04–0.42	0.00
		cTnI	3.17	1.77–5.68	< 0.0001
		CRP	2.16	1.22–3.82	0.01
**Nonfatal AMI**					
Group 3 versus group 4	Men	PCSK9	2.03	1.06–3.91	0.03
		NOx	2.08	1.08–4.00	0.03
	Women	NOx	3.26	1.47–7.23	0.01
		Insulin	2.74	1.23–6.09	0.01
	Men and women	PCSK9	1.67	1.01–2.76	0.05
		NOx	2.60	1.57–4.31	< 0.0001
Group 3 versus group 5	Men	ANGPTL3	2.91	1.49–5.68	< 0.0001
	Women	Leptin	0.24	0.09–0.66	0.01
		cTnI	3.47	1.40–8.64	0.01
		NOx	4.10	1.81–9.31	0.01
		Insulin	5.52	2.00–15.29	0.01
		Total cholesterol	2.55	1.05–6.17	0.04
	Men and women	ANGPTL3	3.29	1.96–5.51	< 0.0001
		cTnI	1.79	1.08–2.98	0.03
		NOx	2.41	1.47–3.96	0.01

PCSK9, proprotein convertase subtilisin/kexin type; ANGPTL3, angiopoietin-like protein 3; NOx, nitrate and nitrite; cTnI, cardiac troponin I; CRP, C-reactive protein; eHR, estimated hazard ratio.

The results indicated that ANGPTL3, cTnI, PCSK9, endothelin, galectin-3, and CRP were associated with cardiovascular death, and ANGPTL3, cTnI, PCSK9, NOx, leptin, insulin, and total cholesterol were associated with nonfatal AMI. ANGPTL3, cTnI, and PCSK9 were not specific and did not discriminate between mortality and nonfatal AMI. Galectin-3, CRP, and endothelin were specific toward cardiovascular death, and leptin, NOx, total cholesterol, and insulin were specific toward nonfatal AMI. CRP, endothelin-1, and galectin-3 were associated with mortality only in men and not in women. PCSK9 and ANGPTL3 were associated with nonfatal AMI in men, and HDL cholesterol, cTnI, insulin, and leptin were associated with nonfatal AMI in women (Table 2).

The data are summarized in Table 3, which lists all models that included 11 specific factors with sex-dependent significant associations. Overall, cTnI appears to be the most impactful sex-independent marker of cardiovascular death (eHR of approximately 3.0), and NOx appeared to have the highest overall impact on nonfatal AMI (eHR of approximately 3.0) that was not associated with sex (Table 3).

**Table 3:** Summary of biomarkers included in stepwise Cox regression analysis for associations with cardiovascular death and nonfatal AMI in men and women.

	Total models	Mean eHR for all models	95%CI (mean for all models)	Pr > ChiSq	Men, models	Women, models	Men and women, models
**Cardiovascular death**							
cTnI	9	3.31	1.56–7.15	0.01	3	3	3
ANGPTL3	5	0.14	0.04–0.59	0.01	2	1	2
CRP	5	3.01	1.49–6.09	0.01	3	0	2
Endothelin	2	0.40	0.19–0.87	0.03	1	0	1
Galectin-3	2	2.01	1.03–3.94	0.04	1	0	1
PCSK9	1	0.52	0.29–0.96	0.04	0	0	1
**Nonfatal AMI**							
NOx	5	2.89	1.48–5.76	0.01	1	2	2
ANGPTL3	2	3.10	1.73–5.60	0.00	1	0	1
cTnI	2	2.63	1.24–5.81	0.02	0	1	1
Insulin	2	4.13	1.61–10.69	0.01	0	2	0
PCSK9	2	1.85	1.03–3.34	0.04	1	0	1
Leptin	1	0.24	0.09–0.66	0.01	0	1	0
Total cholesterol	1	2.55	1.05–6.17	0.04	0	0	1

PCSK9, proprotein convertase subtilisin/kexin type; ANGPTL3, angiopoietin-like protein 3; NOx, nitrate and nitrite; cTnI, cardiac troponin I; CRP, C-reactive protein; eHR, estimated hazard ratio.

### Multivariate Cox regression analysis

Eleven markers that were associated with endpoints in stepwise Cox regression and logistic regression were input into multivariate Cox regression adjusted for age and sex (Table 4).

**Table 4:** Multivariate Cox regression analysis for associations of top 11 biomarkers with cardiovascular deaths and nonfatal AMI adjusted for sex and age.

Comparison	Biomarker	eHR	95% CI	Pr > ChiSq
**Cardiovascular death**				
Group 1 versus group 2	*cTnI*	*2.59*	*1.20–5.60*	*0.02*
	ANGPTL3	0.39	0.11–1.44	0.16
	NOx	1.36	0.72–2.57	0.34
	*CRP*	*2.00*	*1.06–3.76*	*0.03*
	PCSK9	0.71	0.37–1.36	0.30
	Endothelin-1	1.12	0.50–2.48	0.78
	Galectin-3	1.61	0.84–3.10	0.15
	Insulin	0.98	0.43–2.24	0.96
	Total cholesterol	1.51	0.79–2.89	0.21
	Leptin	1.04	0.46–2.35	0.92
Group 1 versus group 5	*cTnI*	*3.44*	*1.71–6.93*	*0.00*
	*ANGPTL3*	*0.16*	*0.05–0.55*	*0.00*
	NOx	1.79	0.96–3.35	0.07
	*CRP*	*2.24*	*1.23–4.10*	*0.01*
	PCSK9	0.89	0.47–1.65	0.70
	Endothelin-1	0.65	0.30–1.42	0.28
	Galectin3	1.62	0.86–3.06	0.13
	Insulin	1.00	0.47–2.12	1.00
	Total cholesterol	1.35	0.72–2.56	0.35
	Leptin	1.28	0.59–2.76	0.53
Group 1 versus group 6	*cTnI*	*3.47*	*1.77–6.81*	*0.00*
	*ANGPTL3*	*0.10*	*0.03–0.33*	*0.00*
	NOx	1.50	0.83–2.71	0.18
	*CRP*	*2.27*	*1.25–4.14*	*0.01*
	PCSK9	0.88	0.48–1.64	0.69
	*Endothelin-1*	*0.42*	*0.20–0.91*	*0.03*
	Galectin3	1.56	0.86–2.85	0.15
	Insulin	0.93	0.46–1.88	0.83
	Total cholesterol	1.29	0.68–2.42	0.44
	Leptin	1.28	0.62–2.65	0.51
**Nonfatal AMI**				
Group 3 versus group 4	*cTnI*	*1.97*	*1.11–3.50*	*0.02*
	ANGPTL3	0.92	0.52–1.63	0.78
	*NOx*	*3.05*	*1.75–5.30*	*0.00*
	CRP	1.12	0.63–2.00	0.69
	PCSK9	1.72	0.98–3.02	0.06
	Endothelin-1	0.84	0.46–1.54	0.58
	Galectin3	0.94	0.53–1.66	0.83
	Insulin	1.46	0.83–2.54	0.19
	Total cholesterol	1.44	0.83–2.47	0.19
	Leptin	0.88	0.48–1.63	0.69
Group 3 versus group 5	*cTnI*	*1.84*	*1.02–3.33*	*0.04*
	*ANGPTL3*	*1.89*	*1.08–3.28*	*0.02*
	*NOx*	*2.76*	*1.61–4.73*	*0.00*
	CRP	1.06	0.57–1.97	0.84
	PCSK9	1.21	0.70–2.10	0.49
	Endothelin	1.30	0.70–2.40	0.40
	Galectin-3	1.02	0.57–1.81	0.94
	Insulin	1.41	0.78–2.54	0.26
	Total cholesterol	1.59	0.92–2.73	0.09
	Leptin	0.71	0.39–1.28	0.25

Significant values are in [italics].

PCSK9, proprotein convertase subtilisin/kexin type; ANGPTL3, angiopoietin-like protein 3; NOx, nitrate and nitrite; cTnI, cardiac troponin I; CRP, C-reactive protein; eHR, estimated hazard ratio.

The results indicated significant associations of 4 out of top 6 biomarkers, which were associated with mortality according to stepwise regression (Table 3), including cTnI, ANGPTL3, CRP, and endothelin-1. Associations with nonfatal AMI were confirmed for 3 out of 7 top biomarkers, including NOx, ANGPTL3, and cTnI. The case–control design of the present study implied the lack of meaningful associations of age or sex with the endpoints (P > 0.05), which was confirmed by Cox regression analysis. Thus, top 6 biomarkers associated with either one of the endpoints were included into another multivariate Cox regression adjusted for age and sex (Table 5).

**Table 5:** Multivariate Cox regression analysis for associations of top 6 biomarkers with cardiovascular death and nonfatal AMI adjusted for sex and age.

Comparison	Biomarker	eHR	95% CI	Pr > ChiSq
**Cardiovascular death**				
Group 1 versus group 2	*cTnI*	*2.93*	*1.62–5.32*	*0.00*
	ANGPTL3	0.42	0.12–1.43	0.16
	NOx	1.31	0.72–2.38	0.38
	*CRP*	*2.31*	*1.27–4.20*	*0.01*
Group 1 versus group 5	*cTnI*	*3.74*	*2.07–6.75*	*0.00*
	*ANGPTL3*	*0.16*	*0.05–0.51*	*0.00*
	*NOx*	*1.83*	*1.02–3.29*	*0.04*
	*CRP*	*2.42*	*1.34–4.38*	*0.00*
Group 1 versus group 6	*cTnI*	*3.55*	*1.96–6.42*	*0.00*
	*ANGPTL3*	*0.09*	*0.03–0.29*	*0.00*
	NOx	1.69	0.94–3.01	0.08
	*CRP*	*2.15*	*1.20–3.87*	*0.01*
**Nonfatal AMI**				
Group 3 versus group 4	*cTnI*	*1.78*	*1.05–3.02*	*0.03*
	ANGPTL3	1.17	0.69–1.99	0.56
	*NOx*	*2.67*	*1.61–4.42*	*0.00*
	CRP	1.14	0.67–1.93	0.63
Group 3 versus group 5	*cTnI*	*1.85*	*1.09–3.14*	*0.02*
	*ANGPTL3*	*2.07*	*1.23–3.51*	*0.01*
	*NOx*	*2.42*	*1.46–3.99*	*0.00*
	CRP	1.20	0.70–2.06	0.51

Significant values are in [italics].

ANGPTL3, angiopoietin-like protein 3; NOx, nitrate and nitrite; cTnI, cardiac troponin I; CRP, C-reactive protein; eHR, estimated hazard ratio.

The results indicated independent associations of 4 biomarkers with cardiovascular death, including cTnI, NOx, CRP, and ANGPTL3. Nonfatal AMI was independently associated with cTnI, NOx, and ANGPTL3. Notably, higher levels of ANGPTL3 were associated with a lower risk of cardiovascular death and a higher risk of nonfatal AMI.

ROC analysis was thus used to resolve this apparent controversy in the case of ANGPTL3 and further confirm potential applicability of detected associations in actual patient stratification at baseline. The results of ROC analysis of associations of biomarkers with pre-existing manifestations of acute coronary syndrome at baseline are shown in Supplemental Table S6, indicating that cTnI, NOx, creatinine, and HDL cholesterol had significant associations and ANGPTL3 was not associated with these manifestations.

## Discussion

Overall, the results of statistical analysis indicated that cTnI was reliably associated with the outcomes of the present study, including cardiovascular mortality and AMI (Tables 4, 5), and can thus be used as a reference to confirm the associations of other experimental biomarkers with the outcomes. The data are in agreement with multiple results of other studies that demonstrated significant associations of cTnI with AMI prognosis^18,20–22^ and cardiovascular mortality^23^. An increase in the circulating levels of cTnI is caused by myocardial injury and release of myocardial proteins from dead cardiomyocytes induced by various processes^24^. An increase in cTnI may be detected in patients with other clinical conditions without obvious connection to cardiac injury; however, higher cTnI levels are associated with poor prognosis regardless of the underlying pathology^25^.

Similar to the data on cTnI, the results of the present study confirmed sex-independent associations of the outcomes with CRP, which is another extensively validated marker. CRP is considered one of the main markers for inflammation, and various cardiovascular diseases are linked to inflammatory processes, contributing to acute cardiovascular events and mortality^26^. Interestingly, the results of the present study did not confirm the associations of CRP with AMI in multivariate Cox regression analysis (Table 5) and in univariate analysis of the associations of CRP with acute coronary syndrome at baseline anamnesis (Supplemental Table S6); however, CRP was independently associated with cardiovascular death based on the results of Cox regression analysis adjusted for age and sex (Table 5).

The levels of NOx have been recently extensively characterized by us and other authors as independent predictors of cardiovascular mortality and acute cardiovascular events, including follow up studies for 3^27^ and 8 years^28^ in a Moscow cohort, a relatively small study in elderly patients in Japan^29^, a Framingham offspring study^30^, and a similar study in Iran^31^. The results of the present study indicated independent associations of high levels of NOx with risk for AMI in all tested models (Tables 4, 5). The molecular mechanisms of these associations remain largely speculative. The levels of NOx in the serum are an integral parameter characterizing various dynamic processes, including excretion of nitrite and nitrate, synthesis and oxidation of endogenous NO, redox reactions, and consumption of exogenous nitrates and nitrites^32^. Moreover, our previous study^27^ and similar analysis of the data obtained in the present study indicated that the levels of NOx were not correlated with other biomarkers, including inflammatory marker CRP (data not shown). An increase in the circulating levels of NOx may be caused by enhanced overall production of NO, which may compensate for a decrease in normal bioavailability of endogenous NO^33^.

Notably, the present study is the first to directly compare the characteristics of NOx as a biomarker with the features of known reliable biomarkers, including cTnI and CRP. The data indicated that high levels of NOx may have better predictive power (eHR of approximately 2.5) for AMI than that of cTnI (eHR of approximately 1.8).

ANGPTL3 is another experimental biomarker that demonstrated consistent associations in the present study. Interestingly, high levels of ANGPTL3 were associated with a reduction in cardiovascular death and an increase in AMI, and these associations persisted after the adjustment for age and sex. Moreover, ANGPTL3 was not associated with baseline cardiovascular diseases (Supplemental Table S6). To the best of our knowledge, long-term associations of ANGPTL3 with cardiovascular mortality have not been investigated. However, a recent study reported a reduction in AMI incidence in subjects with low ANGPTL3 concentrations^34^, which is in general agreement with the data of the present study. This association may be due to proangiogenic activity of ANGPTL3, which is expected to benefit revascularization in coronary circulation^35^ thus promoting the survival of patients with AMI. This result raises an important concern of the general safety of inactivation of ANGPTL3 in patients with coronary artery disease or AMI since ANGPTL3 is considered a novel pharmacological target for the treatment of cardiovascular diseases^36,37^. Rare variants of ANGPTL3 are associated with decreased levels of LDL and HDL cholesterol^38,39^. Thus, the results of the present study indicated that high levels of ANGPTL3 promoted the survival of patients with AMI and are thus generally consistent with complex effects of ANGPTL3^39^.

PCSK9 is involved in the degradation of the LDL receptor and is a novel and potentially important drug target for therapy of cardiovascular diseases^40^. Preclinical data indicate that PCSK9 is upregulated in ischemia in the heart, and a decrease in PCSK9 expression may influence cardiac injury and subsequent morphological and functional remodeling. Clinical data suggest that inhibition of PCSK9 may be associated with a reduction in incidence of acute cardiovascular events in subjects with increased cardiovascular risk^41^. Circulating levels of PCSK9 were shown to be increase in an AMI model in rats^42^ and in cardiomyocytes subjected to hypoxia/reoxygenation^43^. The results of the present study indicated that PCSK9 was associated with AMI and cardiovascular death, although these associations were only borderline significant and could not be confirmed by Cox regression or extended comparison, which may be due to limitations imposed by the design of the present study and relatively low number of participants. Alternatively, PCSK9 may be a dependent parameter. Notably, the associations of high levels of PCSK9 were similar to those detected in the case of ANGPTL3, including a higher risk of AMI and a lower risk of cardiovascular death. Thus, high levels of PCSK9 may enhance the survival of patients with AMI, and inhibition of PCSK9 may also raise certain concerns similar to the issues that are currently considered for therapeutic use of ANGPTL3.

Leptin was included in the present study since this biomarker is a central regulator of food intake and energy expenditure playing the role of an adipose-specific adipokine^44^. Leptin has been considered a biomarker of cardiovascular diseases but did not pass the validation tests^7^. The results of the present study indicated that high levels of leptin were associated with a lower risk of AMI in women in one of the tested models (Table 4). However, subsequent analysis excluded leptin suggesting that additional testing is required for definitive conclusions.

Galectin-3 has been approved as a supplementary indicator for prognosis of patients with heart failure in 2010^45^ despite certain contradictions in the data of clinical investigations. Galectin-3 can influence atherogenesis by multiple mechanisms, and the results of multivariate regression analysis indicated that galectin-3 is a predictor of cardiovascular mortality along with age, left ventricular ejection fraction, and coronary atherosclerotic burden^46^. Another study demonstrated that galectin-3 is an independent predictor of mortality and development of heart failure in patients with previous AMI^47^. The results of the present study indicated that galectin-3 was associated with cardiovascular death with eHR of approximately 1.9 regardless of sex and was not associated with AMI. However, subsequent multivariate Cox regression did not confirm these associations suggesting insufficient sample size or dependence on other tested factors.

We were also unable to confirm the associations of the outcomes with the levels of insulin or total cholesterol. The lack of associations of these parameters may be due to insufficient power of the study, case–control design, low robustness of the markers, or dependence on other tested factors.

Unique design of the present relatively small case–control study enabled comparison of multiple biomarkers to identify independent predictors of cardiovascular mortality and AMI suitable for robust stratification of patients. Thus, the data suggested that subsequent evaluation of prognostic biomarkers in a larger ESSE-RF cohort should involve cTnI, NOx, CRP, and potentially ANGPTL3.

### Limitations

This study has certain limitations that could have influenced the validity of the conclusions. First, like in any case–control study, assessment of the associations by logit regression is inherently biased. Moreover, time-dependent Cox regression analysis cannot produce reliable hazard ratios. Thus, we have used multiple expanded comparisons to confirm the results of logit and Cox regressions obtained by the main case–control comparisons. To estimate the overall potential bias, we have used the bootstrap bias correction function (in R version 4.2.1 with 10,000 iterations). The boot library was used to assess bias-corrected and accelerated bootstrap intervals. The data shown in Supplemental Table S7 indicated that potential bias was very small, thus partially alleviating this concern.

Notably, the main goal of the present study was to compare various biomarkers to each other without an assessment of causal relationships and involved assumption of relatively small and potentially equal bias for all tested parameters^17^. Moreover, the size of the cohort was relatively small and could have been insufficient for the detection of the associations of less robust biomarkers. However, this design also excluded potentially biased and poorly associated biomarkers, thus partially alleviating this concern as well.

Additionally, experimental biomarkers do not have clearly defined reference values, which could have introduced a bias due to the selection of the matched controls. To address this specific concern, a larger randomized study is required.

## Conclusion

Overall results indicated that cardiovascular death was significantly associated with elevated levels of cTnI and CRP, and a similar pattern was detected in the case of NOx in expanded internal confirmation groups. Elevated levels of ANGPTL3 were associated with a higher risk of cardiovascular death and a lower risk of nonfatal AMI. The results confirmed that elevated levels of NOx and cTnI were associated with a higher risk of nonfatal AMI.

The data on associations of various types of risks with cTnI indicated that the study has been properly designed with sufficient power to detect robust associations with the risk for cardiovascular death and nonfatal AMI and can reliably assess potential prognostic impact of various biomarkers. These results require validation in an independent expanded cohort to provide recommendations for nationwide baseline stratification of patients.

## Supplementary Information

Supplementary Information.

## Data Availability

The datasets used and/or analysed during the current study available from the corresponding author on reasonable request.
