# Supplementary material for: Serum biomarkers, including nitric oxide metabolites (NOx), for prognosis of cardiovascular death and acute myocardial infarction in an ESSE-RF case–control cohort with 6.5-year follow up
Source: Sci Rep. 2022 Oct 28;12:18177. doi: 10.1038/s41598-022-22367-x (PMC9616821; doi:10.1038/s41598-022-22367-x)
Supplement: Supplementary file 1 — Supplementary Information. [file 41598_2022_22367_MOESM1_ESM.docx]

Supplementary Data for the manuscript:

**Serum biomarkers, including nitric oxide metabolites (NOx), for prognosis of cardiovascular death and acute myocardial infarction in an ESSE-RF case-control cohort with 6.5-year follow up**

**Nadezdha G. Gumanova*, Natalya L. Bogdanova, Victoria A. Metelskaya, Vladimir I. Tarasov, Alexander Ya. Kots, Vladimir A. Kutsenko, Anna V. Kontsevaya, Oksana M. Drapkina**

National Research Center for Preventive Medicine (NRCPM), Moscow, Russian Federation; 10 Petroverigsky per., building 3, Moscow 101990, Russia.

*****Address correspondence to this author at: Nadezhda G. Gumanova, PhD, Department of Biochemistry, National Research Center for Preventive Medicine, 10 Petroverigsky per., building 3, Moscow 101990. e-mail: gumanova@mail.ru; ngumanova@gnicpm.ru; fax: +7(499)5536851.

**Abbreviations:**

AIC, Akaike information criterion

ANGPTL3, angiopoietin-like protein 3

AMI, acute myocardial infarction

ChiSq, chi-squared test

Chol, cholesterol

CLSI, Clinical Laboratory Standards Institute

CRP, C-reactive protein

cTnI, cardiac troponin I

eHR, estimated hazard ratio

ESSE-RF, Cardiovascular Epidemiology in Russian Federation”

HDL, high density lipoprotein

LDL, low density lipoprotein

NOx, serum nitric oxide metabolites

NRCPM, National Research Center for Preventive Medicine

PCSK9, proprotein convertase subtilisin/kexin type

ROC, receiver operating characteristic

SC, Schwartz criterion

SCORE, Systematic Coronary Risk Estimation

WHO, World Health Organization

| Groups | N | Age (years) | Height (cm) | Weight (kg) | BMI (kg/m^2^) | SBP (mm Hg) | DBP (mm Hg) | Smoking (score) |
| --- | --- | --- | --- | --- | --- | --- | --- | --- |
| *Men* | | | | | | | | |
| 1, case, cardiovascular deaths | 30 | 55 (36-64) | 173 (159-191) | 81.9 (54.9-131.6) | 27.3 (19.5-41.0) | 144 (107-240) | 88 (68-128) | 3 (1-3) |
| 2, matched control to group 1 | 30 | 54 (36-63) | 174 (158-191) | 79.1 (49.0-112.1) | 26.5 (19.3-41.1) | 136 (110-174) | 85 (68-109) | 2 (1-3) |
| 3, case, nonfatal AMI | 37 | 57 (39-63) | 174 (163-187) | 82.2 (59.3-143) | 27.8 (21.5-40.9) | 140 (105-179) | 83 (61-103) | 2 (1-3) |
| 4, matched control to group 3 | 37 | 57 (39-63) | 175 (157-184) | 82 (58-117) | 27.7 (20.9-37.5) | 144 (118-179) | 87 (59-114) | 2 (1-3) |
| *Women* | | | | | | | | |
| 1, case, cardiovascular deaths | 18 | 61 (36-65) | 159 (153-174) | 81.4 (59.0-106.7) | 31.4 (24.0-43.6) | 138 (103-167) | 84 (65-94) | 1 (1-3) |
| 2, matched control to group 1 | 18 | 60 (35-64) | 160 (151-172) | 76.9 (54.5-113.3) | 30.1 (19.3-40.2) | 143 (121-182) | 89 (65-98) | 1 (1-3) |
| 3, case, nonfatal AMI | 26 | 57 (33-64) | 161 (146-171) | 79.1 (44.9-124.9) | 29.7 (19.2-50.7) | 153 (99-200) | 82 (65-104) | 1 (1-3) |
| 4, matched control to group 3 | 26 | 58 (34-64) | 159 (148-172) | 71.3 (54.2-107.1) | 27.6 (20.6-42.5) | 142 (117-193)0 | 84 (64-101) | 1 (1-3) |
| *Men and women* | | | | | | | | |
| 1, case, cardiovascular deaths | 48 | 57 (36-65) | 168 (153-191) | 81.9 (54.9-131.6) | 29.1 (19.5-43.6) | 144 (103-240) | 85 (65-128) | 2 (1-3) |
| 2, matched control to group 1 | 48 | 56 (35-64) | 169 (151-191) | 79.1 (49.0-113.3) | 27.7 (19.3-41.1) | 140 (110-182) | 88 (65-109) | 2 (1-3) |
| 3, case, nonfatal AMI | 63 | 57 (33-64) | 168 (146-187) | 81.0 (44.9-143.0) | 28.1 (19.2-50.7) | 143 (99-200) | 82 (61-104) | 2 (1-3) |
| 4, matched control to group 3 | 63 | 57 (34-64) | 167 (148-184) | 80.0 (54.2-117.4) | 27.7 (20.6-42.5) | 144 (117-193) | 85 (59-114) | 2 (1-3) |
| Total cohort | 222 | 57 (33-65) | 168 (146-191) | 80.6 (44.9-143.0) | 27.9 (19.2-50.7) | 142 (99-240) | 85 (59-128) | 2 (1-3) |

**Supplementary Table S1.** Baseline demographics and characteristics of the total cohort including the case-control groups. The data are shown as median (range). BMI, body mass index; SBP, systolic blood pressure; DBP, diastolic blood pressure; AMI, acute myocardial infarction. Smoking status: never smoked (1), quitted (2), and actual smoker (3).

| Parameter | Leptin | Adiponectin | Endothelin-1 | PCSK9 | Galectin-3 | ANGPTL3 | NOx | cTnI | Total chol | HDL chol | LDL chol | Glucose | Insulin | CRP | | Creatinine | |
| --- | --- | --- | --- | --- | --- | --- | --- | --- | --- | --- | --- | --- | --- | --- | --- | --- | --- |
| Units | ng/mL | µg/mL | pg/mL | ng/mL | ng/mL | ng/mL | µM | pg/mL | mM | mM | mM | mM | µIU/mL | mg/L | | µM | |
| *Men* | | | | | | | | | | | | | | | | | |
| Group 1; N=30 | | | | | | | | | | | | | | | | |  |
| Мedian | 6.64 | 7.70 | 0.81 | 389.24 | 6.84 | 428.82 | 24.58 | 7.30 | 5.31 | 1.13 | 3.53 | 5.53 | 9.25 | 2.36 | 79.65 | |  |
| Мin | 0.77 | 3.75 | 0.08 | 223.96 | 4.18 | 273.11 | 4.89 | 0.30 | 3.40 | 0.67 | 1.48 | 4.06 | 1.30 | 0.24 | 60.70 | |  |
| Max | 30.27 | 62.79 | 1.39 | 800.00 | 9.63 | 709.65 | 148.98 | 147.00 | 10.00 | 2.44 | 7.58 | 28.00 | 23.70 | 84.56 | 286.20 | |  |
| Group 2; N=30 | | | | | | | | | | | | | | | | |  |
| Мedian | 7.86 | 7.82 | 0.75 | 800.00 | 4.97 | 417.72 | 18.55 | 1.40 | 5.16 | 1.31 | 3.25 | 5.56 | 8.25 | 1.13 | 76.35 | |  |
| Мin | 0.07 | 3.97 | 0.08 | 184.20 | 2.46 | 256.95 | 1.96 | 0.30 | 3.14 | 0.80 | 1.56 | 4.14 | 3.30 | 0.23 | 60.00 | |  |
| Max | 52.99 | 22.95 | 2.01 | 800.00 | 10.55 | 836.04 | 115.89 | 17.40 | 7.20 | 2.25 | 5.65 | 6.66 | 22.90 | 4.12 | 96.70 | |  |
| Group 3; N=37 | | | | | | | | | | | | | | | | |  |
| Мedian | 9.39 | 9.95 | 0.99 | 800.00 | 5.98 | 670.92 | 27.54 | 3.00 | 5.23 | 1.24 | 3.45 | 5.38 | 7.50 | 1.24 | 76.50 | |  |
| Мin | 1.19 | 2.43 | 0.08 | 173.46 | 3.55 | 367.67 | 0.05 | 0.90 | 2.74 | 0.79 | 1.39 | 3.20 | 2.70 | 0.26 | 57.00 | |  |
| Max | 49.26 | 52.31 | 2.25 | 800.00 | 12.48 | 1111.89 | 190.83 | 446.40 | 8.18 | 1.82 | 6.45 | 7.60 | 44.50 | 16.49 | 120.20 | |  |
| Group 4; N=37 | | | | | | | | | | | | | | | | |  |
| Мedian | 8.73 | 9.58 | 0.96 | 353.22 | 5.58 | 647.40 | 15.03 | 1.80 | 5.60 | 1.23 | 3.39 | 5.46 | 7.90 | 1.60 | 76.70 | |  |
| Мin | 0.37 | 3.24 | 0.08 | 179.05 | 3.40 | 315.65 | 2.59 | 0.10 | 3.50 | 0.80 | 2.06 | 4.04 | 2.20 | 0.19 | 59.40 | |  |
| Max | 61.51 | 114.87 | 2.10 | 800.00 | 16.33 | 1203.62 | 145.04 | 27.80 | 7.47 | 1.85 | 5.52 | 7.45 | 22.50 | 8.32 | 109.70 | |  |
| All male ; N=134 | | | | | | | | | | | | | | | | |  |
| Мedian | 8.61 | 8.28 | 0.80 | 395.83 | 5.78 | 536.83 | 18.55 | 2.50 | 5.28 | 1.23 | 3.41 | 5.46 | 7.95 | 1.56 | 77.10 | |  |
| Мin | 0.07 | 2.43 | 0.08 | 173.46 | 2.46 | 256.95 | 0.05 | 0.10 | 2.74 | 0.67 | 1.39 | 3.20 | 1.30 | 0.19 | 57.00 | |  |
| Max | 61.51 | 114.87 | 2.25 | 800.00 | 16.33 | 1203.62 | 190.83 | 446.40 | 10.00 | 2.44 | 7.58 | 28.00 | 44.50 | 84.56 | 286.20 | |  |
| *Women* | | | | | | | | | | | | | | | | |  |
| Group 1; N=18 | | | | | | | | | | | | | | | | |  |
| Мedian | 27.56 | 10.27 | 0.77 | 587.05 | 6.04 | 495.98 | 20.25 | 3.85 | 5.52 | 1.35 | 3.30 | 5.53 | 10.80 | 2.32 | 70.55 | |  |
| Мin | 0.49 | 3.29 | 0.08 | 240.82 | 4.05 | 247.14 | 6.50 | 0.50 | 2.94 | 0.75 | 1.76 | 4.36 | 3.20 | 0.73 | 57.20 | |  |
| Max | 52.28 | 39.36 | 3.03 | 800.00 | 9.63 | 780.14 | 88.29 | 31.10 | 7.61 | 1.94 | 5.64 | 16.16 | 199.70 | 31.89 | 88.40 | |  |
| Group 2; N=18 | | | | | | | | | | | | | | | | |  |
| Мedian | 35.37 | 11.94 | 0.62 | 800.00 | 6.02 | 481.30 | 22.26 | 1.45 | 5.98 | 1.49 | 4.15 | 5.53 | 9.00 | 2.53 | 66.10 | |  |
| Мin | 12.02 | 3.17 | 0.08 | 299.98 | 3.04 | 272.87 | 0.18 | 0.10 | 3.99 | 1.15 | 2.24 | 3.11 | 4.80 | 0.22 | 55.30 | |  |
| Max | 58.52 | 26.04 | 1.37 | 800.00 | 11.07 | 975.69 | 60.87 | 10.00 | 7.65 | 2.10 | 5.17 | 6.09 | 14.90 | 20.20 | 78.00 | |  |
| Group 3; N=26 | | | | | | | | | | | | | | | | |  |
| Мedian | 30.98 | 8.07 | 1.07 | 800.00 | 7.85 | 794.96 | 28.46 | 2.60 | 6.66 | 1.50 | 4.01 | 5.52 | 12.55 | 2.16 | 69.05 | |  |
| Мin | 2.05 | 2.88 | 0.08 | 271.92 | 3.26 | 521.99 | 3.36 | 0.40 | 4.87 | 1.07 | 2.28 | 3.86 | 3.40 | 0.27 | 52.10 | |  |
| Max | 70.80 | 113.29 | 1.81 | 800.00 | 10.77 | 1226.66 | 99.23 | 18.40 | 9.62 | 2.44 | 6.97 | 10.32 | 54.50 | 12.82 | 254.90 | |  |
| Group 4; N=26 | | | | | | | | | | | | | | | | |  |
| Мedian | 22.79 | 10.89 | 0.69 | 800.00 | 7.24 | 760.59 | 10.62 | 1.85 | 5.65 | 1.49 | 3.61 | 5.08 | 6.30 | 1.73 | 62.70 | |  |
| Мin | 5.04 | 3.44 | 0.08 | 277.02 | 0.03 | 310.68 | 0.05 | 0.10 | 4.16 | 1.03 | 0.56 | 4.37 | 3.30 | 0.16 | 51.70 | |  |
| Max | 73.70 | 44.16 | 1.75 | 800.00 | 10.33 | 1275.31 | 56.52 | 4.20 | 8.49 | 2.67 | 5.91 | 20.80 | 53.50 | 11.60 | 86.10 | |  |
| All female; N=88 | | | | | | | | | | | | | | | | |  |
| Мedian | 28.70 | 10.09 | 0.71 | 800.00 | 6.91 | 645.62 | 20.54 | 2.00 | 6.11 | 1.46 | 3.85 | 5.48 | 9.40 | 2.17 | 66.30 | |  |
| Мin | 0.49 | 2.88 | 0.08 | 240.82 | 0.03 | 247.14 | 0.05 | 0.10 | 2.94 | 0.75 | 0.56 | 3.11 | 3.20 | 0.16 | 51.70 | |  |
| Max | 73.70 | 113.29 | 3.03 | 800.00 | 11.07 | 1275.31 | 99.23 | 31.10 | 9.62 | 2.67 | 6.97 | 20.80 | 199.70 | 31.89 | 254.90 | |  |
| *Men and women* | | | | | | | | | | | | | | | | |  |
| Group 1; N=48 | | | | | | | | | | | | | | | | |  |
| Мedian | 14.67 | 8.90 | 0.78 | 389.24 | 6.66 | 444.66 | 23.57 | 4.25 | 5.41 | 1.17 | 3.39 | 5.53 | 10.00 | 2.32 | 73.80 | |  |
| Мin | 0.49 | 3.29 | 0.08 | 223.96 | 4.05 | 247.14 | 4.89 | 0.30 | 2.94 | 0.67 | 1.48 | 4.06 | 1.30 | 0.24 | 57.20 | |  |
| Max | 52.28 | 62.79 | 3.03 | 800.00 | 9.63 | 780.14 | 148.98 | 147.00 | 10.00 | 2.44 | 7.58 | 28.00 | 199.70 | 84.56 | 286.20 | |  |
| Group 2; N=48 | | | | | | | | | | | | | | | | |  |
| Мedian | 15.25 | 8.74 | 0.69 | 800.00 | 5.52 | 429.86 | 19.87 | 1.40 | 5.43 | 1.37 | 3.56 | 5.56 | 8.40 | 1.23 | 72.85 | |  |
| Мin | 0.07 | 3.17 | 0.08 | 184.20 | 2.46 | 256.95 | 0.18 | 0.10 | 3.14 | 0.80 | 1.56 | 3.11 | 3.30 | 0.22 | 55.30 | |  |
| Max | 58.52 | 26.04 | 2.01 | 800.00 | 11.07 | 975.69 | 115.89 | 17.40 | 7.65 | 2.25 | 5.65 | 6.66 | 22.90 | 20.20 | 96.70 | |  |
| Group 3; N=63 | | | | | | | | | | | | | | | | |  |
| Мedian | 16.83 | 8.30 | 1.01 | 800.00 | 6.62 | 709.34 | 27.54 | 2.90 | 5.91 | 1.31 | 3.70 | 5.38 | 8.60 | 1.66 | 73.60 | |  |
| Мin | 1.19 | 2.43 | 0.08 | 173.46 | 3.26 | 367.67 | 0.05 | 0.40 | 2.74 | 0.79 | 1.39 | 3.20 | 2.70 | 0.26 | 52.10 | |  |
| Max | 70.80 | 113.29 | 2.25 | 800.00 | 12.48 | 1226.66 | 190.83 | 446.40 | 9.62 | 2.44 | 6.97 | 10.32 | 54.50 | 16.49 | 254.90 | |  |
| Group 4; N=63 | | | | | | | | | | | | | | | | |  |
| Мedian | 12.26 | 10.13 | 0.88 | 393.85 | 6.30 | 670.92 | 14.05 | 1.80 | 5.60 | 1.32 | 3.46 | 5.31 | 7.30 | 1.63 | 70.60 | |  |
| Мin | 0.37 | 3.24 | 0.08 | 179.05 | 0.03 | 310.68 | 0.05 | 0.10 | 3.50 | 0.80 | 0.56 | 4.04 | 2.20 | 0.16 | 51.70 | |  |
| Max | 73.70 | 114.87 | 2.10 | 800.00 | 16.33 | 1275.31 | 145.04 | 27.80 | 8.49 | 2.67 | 5.91 | 20.80 | 53.50 | 11.60 | 109.70 | |  |
| *Total cohort; N=222* | | | | | | | | | | | | | | | | |  |
| Мedian | 14.60 | 8.83 | 0.79 | 800.00 | 6.25 | 578.63 | 19.73 | 2.30 | 5.56 | 1.31 | 3.51 | 5.47 | 8.40 | 1.68 | 72.95 | |  |
| Мin | 0.07 | 2.43 | 0.08 | 173.46 | 0.03 | 247.14 | 0.05 | 0.10 | 2.74 | 0.67 | 0.56 | 3.11 | 1.30 | 0.16 | 51.70 | |  |
| Max | 73.70 | 114.87 | 3.03 | 800.00 | 16.33 | 1275.31 | 190.83 | 446.40 | 10.00 | 2.67 | 7.58 | 28.00 | 199.70 | 84.56 | 286.20 | |  |
| Reference range |  | NA | NA | NA | NA | NA | NA | <26.2 | <4.9 | >1.0 men, >1.2 women | <3.0 | <6.1 | 2-22 | <3.0 | 74.3-107 | |  |

**Supplementary Table S2.** Biochemical markers in the cohort. PCSK9, proprotein convertase subtilisin/kexin type; ANGPTL3, angiopoietin-like protein 3; NOx, nitrate and nitrite; cTnI, cardiac troponin I; chol, cholesterol; HDL, high density lipoproteins; LDL, low density lipoproteins; CRP, C-reactive protein. NA, reference range for experimental biomarkers is not defined.

| Biomarker | Units | Men | Women | Men and women |
| --- | --- | --- | --- | --- |
| cTnI | pg/mL | 3.90 | 2.90 | 3.40 |
| NOx | µM | 29.42 | 29.67 | 29.42 |
| ANGPTL3 | ng/mL | 630.99 | 759.63 | 670.92 |
| PCSK9 | ng/mL | 800.00 | 800.00 | 800.00 |
| Galectin-3 | ng/mL | 6.74 | 7.49 | 7.22 |
| Leptin | ng/mL | 13.71 | 38.02 | 22.59 |
| Adiponectin | µg/mL | 11.28 | 13.31 | 11.89 |
| Endothelin | pg/mL | 1.01 | 1.10 | 1.03 |
| CRP | mg/L | 2.08 | 4.5 | 2.44 |
| Creatinine | µM | 81.6 | 70.6 | 77.4 |
| Total cholesterol | mM | 5.85 | 6.7 | 6.22 |
| LDL cholesterol | mM | 3.86 | 4.51 | 4.12 |
| HDL cholesterol | mM | 1.34 | 1.61 | 1.46 |
| Glucose | mM | 5.75 | 5.73 | 5.75 |
| Insulin | µIU/mL | 9.70 | 11.70 | 10.80 |

**Supplementary Table S3.** Cutoff concentrations of biomarkers for upper tercile in the total cohort, in men, and in women. PCSK9, proprotein convertase subtilisin/kexin type; ANGPTL3, angiopoietin-like protein 3; NOx, nitrate and nitrite; cTnI, cardiac troponin I; HDL, high density lipoproteins; LDL, low density lipoproteins; CRP, C-reactive protein.

| Biomarker | Men | | | | Women | | | | Men and women | | | |
| --- | --- | --- | --- | --- | --- | --- | --- | --- | --- | --- | --- | --- |
|  | OR | 95% CI | ROC | Pr > ChiSq | OR | 95% CI | ROC | Pr > ChiSq | OR | 95% CI | ROC | Pr > ChiSq |
| Group 1 versus group 2 | | | | | | | | | | | | |
| *Experimental biomarkers* | | | | | | | | | | | | |
| Leptin | 1.35 | 0.46-3.97 | 0.53 | 0.58 | 0.29 | 0.07-1.21 | 0.64 | 0.09 | 0.91 | 0.39-2.12 | 0.51 | 0.83 |
| Adiponectin | 1.46 | 0.44-4.86 | 0.53 | 0.54 | 0.79 | 0.20-3.07 | 0.53 | 0.73 | 1.11 | 0.46-2.70 | 0.51 | 0.82 |
| Endothelin | 1.98 | 0.51-7.64 | 0.55 | 0.32 | 1.00 | 0.17-5.77 |  | 1.00 | 1.54 | 0.53-4.46 | 0.53 | 0.42 |
| PCSK9 | 0.59 | 0.21-1.62 | 0.57 | 0.30 | 0.39 | 0.10-1.54 | 0.61 | 0.18 | 0.51 | 0.23-1.15 | 0.58 | 0.10 |
| Galectin-3 | 3.76 | 1.24-11.39 | 0.65 | 0.02 | 3.08 | 0.51-18.54 | 0.58 | 0.22 | 2.84 | 1.12-7.18 | 0.60 | 0.03 |
| ANGPTL3 | 1.00 | 0.13-7.61 |  | 1.00 | 1.00 | 0.06-17.33 |  | 1.00 | 1.53 | 0.25-9.61 | 0.51 | 0.65 |
| cTnI | 5.68 | 1.84-17.49 | 0.70 | 0.01 | 6.25 | 1.33-29.43 | 0.69 | 0.02 | 5.13 | 2.11-12.47 | 0.69 | 0.01 |
| NOx | 2.10 | 0.71-6.22 | 0.58 | 0.18 | 2.08 | 0.52-8.34 | 0.58 | 0.30 | 2.09 | 0.89-4.92 | 0.58 | 0.09 |
| *Routine biomarkers* | | | | | | | | | | | | |
| CRP | 6.54 | 1.97-21.74 | 0.70 | 0.01 | 0.77 | 0.19-3.19 | 0.53 | 0.72 | 2.28 | 0.97-5.35 | 0.59 | 0.06 |
| Creatinine | 2.10 | 0.71-6.22 | 0.58 | 0.18 | 2.60 | 0.65-10.38 | 0.61 | 0.18 | 1.71 | 0.74-3.95 | 0.56 | 0.21 |
| Total chol | 2.67 | 0.84-8.46 | 0.60 | 0.10 | 0.57 | 0.13-2.51 | 0.56 | 0.46 | 1.85 | 0.75-4.52 | 0.56 | 0.18 |
| LDL chol | 3.06 | 0.97-9.66 | 0.62 | 0.06 | 0.60 | 0.15-2.45 | 0.56 | 0.48 | 1.00 | 0.42-2.37 |  | 1.00 |
| HDL choll | 0.25 | 0.08-0.79 | 0.65 | 0.02 | 0.74 | 0.16-3.38 | 0.53 | 0.70 | 0.50 | 0.20-1.21 | 0.57 | 0.12 |
| Glucose | 1.15 | 0.41-3.20 | 0.52 | 0.79 | 2.23 | 0.52-9.59 | 0.58 | 0.28 | 1.31 | 0.57-3.02 | 0.53 | 0.53 |
| Insulin | 2.04 | 0.71-5.90 | 0.58 | 0.19 | 3.18 | 0.67-15.15 | 0.61 | 0.15 | 1.74 | 0.74-4.05 | 0.56 | 0.20 |
| Group 1 versus group 5 | | | | | | | | | | | | |
| *Experimental biomarkers* | | | | | | | | | | | | |
| Leptin | 1.27 | 0.51-3.13 | 0.53 | 0.61 | 0.45 | 0.13-1.61 | 0.58 | 0.22 | 1.04 | 0.51-2.14 | 0.50 | 0.91 |
| Adiponectin | 0.74 | 0.29-1.94 | 0.53 | 0.54 | 0.72 | 0.23-2.28 | 0.54 | 0.58 | 0.73 | 0.35-1.52 | 0.53 | 0.40 |
| Endothelin | 0.67 | 0.25-1.80 | 0.54 | 0.42 | 0.43 | 0.11-1.73 | 0.58 | 0.23 | 0.57 | 0.26-1.28 | 0.55 | 0.17 |
| PCSK9 | 0.89 | 0.37-2.11 | 0.51 | 0.79 | 0.52 | 0.17-1.58 | 0.58 | 0.25 | 0.72 | 0.37-1.42 | 0.54 | 0.34 |
| Galectin-3 | 3.11 | 1.27-7.64 | 0.63 | 0.01 | 1.03 | 0.30-3.50 | 0.50 | 0.97 | 1.62 | 0.80-3.29 | 0.55 | 0.18 |
| ANGPTL3 | 0.15 | 0.03-0.67 | 0.63 | 0.01 | 0.11 | 0.01-0.94 | 0.64 | 0.04 | 0.15 | 0.04-0.52 | 0.62 | 0.01 |
| cTnI | 5.99 | 2.34-15.31 | 0.70 | 0.01 | 5.63 | 1.69-18.76 | 0.69 | 0.01 | 5.53 | 2.66-11.53 | 0.69 | <.0001 |
| NOx | 2.44 | 0.98-6.09 | 0.60 | 0.06 | 3.11 | 0.95-10.16 | 0.62 | 0.06 | 2.68 | 1.30-5.52 | 0.61 | 0.01 |
| *Routine biomarkers* | | | | | | | | | | | | |
| CRP | 3.85 | 1.55-9.54 | 0.66 | 0.01 | 0.92 | 0.27-3.10 | 0.51 | 0.89 | 2.09 | 1.04-4.21 | 0.59 | 0.04 |
| Creatinine | 2.08 | 0.85-5.13 | 0.58 | 0.11 | 3.40 | 1.06-10.87 | 0.64 | 0.04 | 1.92 | 0.95-3.88 | 0.57 | 0.07 |
| Total chol | 1.46 | 0.60-3.57 | 0.54 | 0.41 | 0.68 | 0.19-2.47 | 0.54 | 0.56 | 1.30 | 0.63-2.66 | 0.53 | 0.48 |
| LDL chol | 1.80 | 0.74-4.38 | 0.57 | 0.20 | 0.82 | 0.25-2.77 | 0.52 | 0.75 | 0.84 | 0.41-1.73 | 0.52 | 0.64 |
| HDL chol | 0.35 | 0.13-0.96 | 0.61 | 0.04 | 0.50 | 0.14-1.78 | 0.57 | 0.28 | 0.47 | 0.22-1.02 | 0.58 | 0.06 |
| Glucose | 1.56 | 0.65-3.79 | 0.55 | 0.32 | 1.91 | 0.59-6.14 | 0.57 | 0.28 | 1.55 | 0.76-3.14 | 0.55 | 0.23 |
| Insulin | 1.92 | 0.79-4.64 | 0.58 | 0.15 | 2.48 | 0.75-8.20 | 0.59 | 0.14 | 1.76 | 0.87-3.57 | 0.56 | 0.12 |
| Group 1 versus group 6 | | | | | | | | | | | | |
| *Experimental biomarkers* | | | | | | | | | | | | |
| Leptin | 1.14 | 0.49-2.66 | 0.52 | 0.76 | 0.48 | 0.14-1.63 | 0.57 | 0.24 | 0.93 | 0.47-1.82 | 0.51 | 0.82 |
| Adiponectin | 0.63 | 0.26-1.56 | 0.55 | 0.32 | 0.96 | 0.32-2.87 | 0.50 | 0.94 | 0.74 | 0.37-1.49 | 0.53 | 0.40 |
| Endothelin | 0.51 | 0.20-1.29 | 0.57 | 0.15 | 0.32 | 0.08-1.20 | 0.61 | 0.09 | 0.43 | 0.20-0.92 | 0.59 | 0.03 |
| PCSK9 | 0.80 | 0.35-1.80 | 0.53 | 0.58 | 0.56 | 0.20-1.58 | 0.57 | 0.27 | 0.69 | 0.36-1.31 | 0.55 | 0.25 |
| Galectin-3 | 2.82 | 1.23-6.49 | 0.62 | 0.01 | 0.65 | 0.21-2.04 | 0.55 | 0.46 | 1.35 | 0.70-2.60 | 0.53 | 0.38 |
| ANGPTL3 | 0.10 | 0.02-0.43 | 0.68 | 0.01 | 0.08 | 0.01-0.66 | 0.68 | 0.02 | 0.09 | 0.03-0.30 | 0.68 | <.0001 |
| cTnI | 4.93 | 2.08-11.67 | 0.69 | 0.01 | 3.13 | 1.08-9.06 | 0.63 | 0.04 | 4.01 | 2.06-7.81 | 0.66 | <.0001 |
| NOx | 1.65 | 0.72-3.78 | 0.56 | 0.24 | 1.75 | 0.61-5.03 | 0.57 | 0.30 | 1.68 | 0.88-3.24 | 0.56 | 0.12 |
| *Routine biomarkers* | | | | | | | | | | | | |
| CRP | 3.38 | 1.46-7.83 | 0.64 | 0.01 | 0.69 | 0.22-2.17 | 0.54 | 0.53 | 1.88 | 0.98-3.61 | 0.57 | 0.06 |
| Creatinine | 1.65 | 0.72-3.78 | 0.56 | 0.24 | 2.33 | 0.81-6.71 | 0.60 | 0.12 | 1.68 | 0.88-3.24 | 0.56 | 0.12 |
| Total chol | 1.37 | 0.59-3.17 | 0.54 | 0.46 | 0.48 | 0.14-1.63 | 0.57 | 0.24 | 1.07 | 0.55-2.09 | 0.51 | 0.85 |
| LDL chol | 1.65 | 0.72-3.78 | 0.56 | 0.24 | 0.65 | 0.21-2.04 | 0.55 | 0.46 | 0.84 | 0.42-1.67 | 0.52 | 0.62 |
| HDL chol | 0.40 | 0.15-1.06 | 0.59 | 0.07 | 0.48 | 0.14-1.63 | 0.57 | 0.24 | 0.49 | 0.23-1.02 | 0.58 | 0.06 |
| Glucose | 1.58 | 0.69-3.61 | 0.55 | 0.28 | 1.30 | 0.45-3.79 | 0.53 | 0.63 | 1.35 | 0.70-2.60 | 0.53 | 0.38 |
| Insulin | 1.88 | 0.82-4.31 | 0.57 | 0.13 | 1.30 | 0.45-3.79 | 0.53 | 0.63 | 1.51 | 0.78-2.90 | 0.55 | 0.22 |

**Supplementary Table S4.** Univariate logistic regression for associations of biomarkers with cardiovascular deaths using binary logit values of the highest tercile versus combined lower terciles and Fisher scoring. PCSK9, proprotein convertase subtilisin/kexin type; ANGPTL3, angiopoietin-like protein 3; NOx, nitrate and nitrite; cTnI, cardiac troponin I; chol, cholesterol; HDL, high density lipoproteins; LDL, low density lipoproteins; CRP, C-reactive protein.

| Biomarker | Men | | | | Women | | | | Men and women | | | |
| --- | --- | --- | --- | --- | --- | --- | --- | --- | --- | --- | --- | --- |
|  | OR | 95% CI | ROC | Pr > ChiSq | OR | 95% CI | ROC | Pr > ChiSq | OR | 95% CI | ROC | Pr > ChiSq |
| Group 3 versus group 4 | | | | | | | | | | | | |
| *Experimental biomarkers* | | | | | | | | | | | | |
| Leptin | 1.27 | 0.49-3.30 | 0.53 | 0.63 | 1.19 | 0.37-3.80 | 0.52 | 0.77 | 1.52 | 0.73-3.19 | 0.55 | 0.26 |
| Adiponectin | 1.00 | 0.40-2.51 |  | 1.00 | 0.41 | 0.12-1.36 | 0.60 | 0.14 | 0.72 | 0.35-1.47 | 0.54 | 0.36 |
| Endothelin | 1.12 | 0.45-2.78 | 0.51 | 0.82 | 1.36 | 0.46-4.07 | 0.54 | 0.58 | 1.21 | 0.60-2.44 | 0.52 | 0.59 |
| PCSK9 | 1.93 | 0.77-4.88 | 0.58 | 0.16 | 1.00 | 0.33-3.06 |  | 1.00 | 1.47 | 0.73-2.96 | 0.55 | 0.29 |
| Galectin-3 | 1.13 | 0.42-3.04 | 0.51 | 0.80 | 1.87 | 0.62-5.63 | 0.58 | 0.27 | 1.14 | 0.56-2.35 | 0.52 | 0.71 |
| ANGPTL3 | 1.25 | 0.50-3.13 | 0.53 | 0.64 | 1.00 | 0.34-2.98 |  | 1.00 | 1.69 | 0.83-3.43 | 0.56 | 0.15 |
| cTnI | 1.74 | 0.61-4.93 | 0.55 | 0.30 | 3.60 | 1.04-12.48 | 0.63 | 0.04 | 2.37 | 1.07-5.24 | 0.59 | 0.03 |
| NOx | 3.08 | 1.12-8.50 | 0.62 | 0.03 | 5.50 | 1.48-20.46 | 0.67 | 0.01 | 3.86 | 1.74-8.60 | 0.64 | 0.01 |
| *Routine biomarkers* | | | | | | | | | | | | |
| CRP | 1.00 | 0.38-2.65 |  | 1.00 | 2.33 | 0.73-7.42 | 0.60 | 0.15 | 1.24 | 0.59-2.62 | 0.52 | 0.57 |
| Creatinine | 1.84 | 0.69-4.90 | 0.57 | 0.22 | 3.08 | 0.88-10.72 | 0.62 | 0.08 | 1.56 | 0.73-3.32 | 0.55 | 0.25 |
| Total cholesterol | 0.80 | 0.31-2.04 | 0.53 | 0.63 | 2.71 | 0.85-8.64 | 0.62 | 0.09 | 1.31 | 0.64-2.69 | 0.53 | 0.46 |
| LDL cholesterol | 0.89 | 0.35-2.29 | 0.51 | 0.81 | 2.33 | 0.73-7.42 | 0.60 | 0.15 | 0.87 | 0.42-1.80 | 0.52 | 0.71 |
| HDL cholesterol | 0.89 | 0.34-2.32 | 0.51 | 0.81 | 0.85 | 0.28-2.58 | 0.52 | 0.78 | 0.87 | 0.43-1.80 | 0.52 | 0.71 |
| Glucose | 1.30 | 0.48-3.52 | 0.53 | 0.61 | 2.33 | 0.73-7.42 | 0.60 | 0.15 | 1.67 | 0.78-3.54 | 0.56 | 0.18 |
| Insulin | 1.00 | 0.38-2.65 |  | 1.00 | 3.89 | 1.18-12.84 | 0.65 | 0.03 | 1.54 | 0.73-3.25 | 0.55 | 0.26 |
| Group 3 versus group 5 | | | | | | | | | | | | |
| *Experimental biomarkers* | | | | | | | | | | | | |
| Leptin | 1.33 | 0.58-3.09 | 0.53 | 0.50 | 0.84 | 0.31-2.31 | 0.52 | 0.74 | 1.37 | 0.72-2.61 | 0.54 | 0.34 |
| Adiponectin | 1.56 | 0.68-3.56 | 0.55 | 0.29 | 0.43 | 0.15-1.29 | 0.59 | 0.13 | 0.95 | 0.50-1.82 | 0.51 | 0.88 |
| Endothelin | 2.08 | 0.91-4.74 | 0.59 | 0.08 | 2.14 | 0.79-5.81 | 0.59 | 0.13 | 2.10 | 1.11-3.97 | 0.59 | 0.02 |
| PCSK9 | 1.37 | 0.61-3.06 | 0.54 | 0.45 | 0.83 | 0.30-2.26 | 0.52 | 0.71 | 1.13 | 0.61-2.11 | 0.52 | 0.69 |
| Galectin-3 | 1.31 | 0.55-3.13 | 0.53 | 0.55 | 3.11 | 1.13-8.60 | 0.63 | 0.03 | 1.62 | 0.85-3.11 | 0.55 | 0.14 |
| ANGPTL3 | 3.00 | 1.31-6.89 | 0.63 | 0.01 | 2.26 | 0.84-6.08 | 0.60 | 0.11 | 3.94 | 2.05-7.56 | 0.66 | <.0001 |
| cTnI | 1.66 | 0.68-4.08 | 0.55 | 0.27 | 3.86 | 1.30-11.44 | 0.64 | 0.01 | 2.23 | 1.13-4.40 | 0.58 | 0.02 |
| NOx | 2.71 | 1.15-6.38 | 0.61 | 0.02 | 3.89 | 1.35-11.25 | 0.65 | 0.01 | 3.13 | 1.61-6.08 | 0.63 | 0.01 |
| *Routine biomarkers* | | | | | | | | | | | | |
| CRP | 1.41 | 0.59-3.41 | 0.54 | 0.44 | 2.04 | 0.75-5.60 | 0.58 | 0.16 | 1.33 | 0.68-2.57 | 0.53 | 0.40 |
| Creatinine | 1.86 | 0.79-4.34 | 0.57 | 0.15 | 2.49 | 0.87-7.12 | 0.60 | 0.09 | 1.42 | 0.74-2.74 | 0.54 | 0.30 |
| Total cholesterol | 1.19 | 0.51-2.78 | 0.52 | 0.69 | 2.38 | 0.87-6.52 | 0.60 | 0.09 | 1.66 | 0.87-3.17 | 0.56 | 0.12 |
| LDL cholesterol | 1.27 | 0.54-2.99 | 0.53 | 0.58 | 1.84 | 0.68-4.99 | 0.57 | 0.23 | 0.99 | 0.52-1.89 | 0.50 | 0.98 |
| HDL cholesterol | 0.67 | 0.29-1.55 | 0.55 | 0.35 | 1.09 | 0.40-2.98 | 0.51 | 0.86 | 0.91 | 0.48-1.72 | 0.51 | 0.77 |
| Glucose | 0.98 | 0.42-2.31 | 0.50 | 0.97 | 2.57 | 0.92-7.20 | 0.61 | 0.07 | 1.46 | 0.76-2.79 | 0.54 | 0.26 |
| Insulin | 1.05 | 0.45-2.49 | 0.51 | 0.91 | 4.54 | 1.57-13.14 | 0.67 | 0.01 | 1.52 | 0.79-2.92 | 0.55 | 0.21 |

**Supplementary Table S5.** Univariate logistic regression of associations of biomarkers with nonfatal AMI using binary logit values of the highest tercile versus combined lower terciles and Fisher scoring. PCSK9, proprotein convertase subtilisin/kexin type; ANGPTL3, angiopoietin-like protein 3; NOx, nitrate and nitrite; cTnI, cardiac troponin I; HDL, high density lipoproteins; LDL, low density lipoproteins; CRP, C-reactive protein.

| Biomarker | AUC | 95%CI | *P* |
| --- | --- | --- | --- |
| cTnI | 0.71 | 0.58-0.84 | 0.00 |
| NOx | 0.65 | 0.52-0.78 | 0.03 |
| PCSK9 | 0.51 | 0.38-0.64 | 0.91 |
| Galectin-3 | 0.48 | 0.36-0.60 | 0.75 |
| ANGPTL3 | 0.47 | 0.34-0.60 | 0.63 |
| Leptin | 0.51 | 0.39-0.64 | 0.84 |
| Adiponectin | 0.50 | 0.37-0.62 | 0.96 |
| Endothelin-1 | 0.57 | 0.44-0.69 | 0.33 |
| Insulin | 0.62 | 0.48-0.75 | 0.09 |
| Glucose | 0.51 | 0.36-0.67 | 0.84 |
| CRP | 0.61 | 0.47-0.75 | 0.10 |
| Creatinine | 0.70 | 0.50-0.80 | 0.01 |
| Total cholesterol | 0.40 | 0.30-0.60 | 0.31 |
| HDL cholesterol | 0.32 | 0.21-0.43 | 0.01 |
| LDL cholesterol | 0.45 | 0.30-0.59 | 0.42 |

**Supplementary Table S6.** ROC analysis of associations of biomarkers with acute coronary syndrome according to baseline anamnesis (N = 27) in the total cohort (N = 222). PCSK9, proprotein convertase subtilisin/kexin type; ANGPTL3, angiopoietin-like protein 3; NOx, nitrate and nitrite; cTnI, cardiac troponin I; CRP, C-reactive protein; HDL, high density lipoproteins; LDL, low density lipoproteins; AUC, area under the curve.

| Parameter | No adjustment | | Bootstrap bias correction | |
| --- | --- | --- | --- | --- |
|  | eHR (Cox regression) | 95CI | eHR (Cox regression) | 95CI |
| Leptin | 1.33 | 0.81-2.20 | 1.32 | 0.79-2.34 |
| Adiponectin | 0.97 | 0.64-1.45 | 0.97 | 0.63-1.45 |
| Endothelin | 1.01 | 0.68-1.49 | 1.01 | 0.68-1.48 |
| PCSK-9 | 1.07 | 0.73-1.57 | 1.06 | 0.72-1.57 |
| Galectin-3 | 1.33 | 0.90-1.96 | 1.33 | 0.90-1.94 |
| ANGPTL3 | 1.47 | 0.98-2.19 | 1.46 | 0.96-2.25 |
| NOx | 1.87* | 1.27-2.73 | 1.86* | 1.25-2.72 |
| cTnI | 2.12* | 1.44-3.12 | 2.10* | 1.40- 3.07 |
| Cortisol | 0.97 | 0.64-1.45 | 0.98 | 0.63-1.47 |
| Lp(a) | 1.48 | 1.01-2.19 | 1.48 | 0.97-2.20 |

**Supplementary Table S7.** Bootstrap bias correction of univariate Cox regression for selected biomarkers. PCSK9, proprotein convertase subtilisin/kexin type; ANGPTL3, angiopoietin-like protein 3; NOx, nitrate and nitrite; cTnI, cardiac troponin I; Lp(a), lipoprotein a; *, P<0.05 for regression.
